# Validity and Reliability of questionnaires measuring attitudes to oral health: A review of the literature

**DOI:** 10.4317/jced.59455

**Published:** 2022-09-01

**Authors:** Rocío del Pilar Ríos-León, Jessica-Margot Salas-Huallparimache, María-Elena Díaz-Pizán, Daniel-José Blanco-Victorio

**Affiliations:** 1 Faculty of Dentistry, Universidad Nacional Mayor de San Marcos. Lima, Perú; 2 Faculty of Health Sciences: (Professional Career of Stomatology). Universidad Científica del Sur. Lima, Perú; 3 Faculty of Stomatology. Universidad Peruana Cayetano Heredia. Lima, Perú; 4 Faculty of Health Sciences, Medical School. Universidad Señor de Sipán, Chiclayo, Perú

## Abstract

**Background:**

The instruments used to assess attitudes to oral health must be validated in order for their results to be reliable and comparable with other variables. The aim of this review was to analyze the ability of self-administered questionnaires to validly and reliably measure attitudes to oral health.

**Material and Methods:**

A bibliographic review was carried out using the following databases, Medline (PubMed), SCOPUS and Web of Science from the year 2016 to 2021, using the keywords: (questionnaire* OR survey*) AND (attitude* OR behav*) AND (“oral health” OR “dental care”) AND (validity OR reliability).

**Results:**

A total of 234 original articles were found in the databases, only 22 met the selection criteria, of which 13 were aimed at patients and nine at health professionals. Evidence of validity and reliability was determined using “COnsensus-based Standards for the selection of health status Measurement INstruments” (COSMIN).

**Conclusions:**

Most of the articles partially meet the validity and reliability criteria.

** Key words:**Questionnaire, attitude, behavior, oral health, validity and reliability.

## Introduction

According to estimates by the World Health Organization (WHO) and the Global Burden of Oral Disease Study 2017, morbidity of oral diseases affects about 3500 billion people worldwide especially in developing countries (1-3). These diseases rank fourth in developed countries and their treatment costs are high (4). Knowing that they are multifactorial, many times only their clinical or restorative aspects are considered and other factors such as social and psychological aspects are not taken in consideration where two main actors are involved: patients and health professionals.

Current health research quantitatively measures psychological factors related to the triad of knowledge, attitudes and practices in oral health (5,6). In some of them, the attitude construct is based on psychological models such as The Theory of Planned Behavior (TPB) and The Health Belief Model (HBM), which have the advantage of evaluating the attitude construct not only in one dimension, but also incorporating other intrinsic factors and thus modifying inappropriate health behaviors (7). Some authors therefore consider this to be one of the main determinants influencing healthy behavior by an individual, rather than the accumulation of knowledge (7,8). In this regard, instruments have been designed to evaluate the attitude construct in other areas of health, taking several dimensions into account (9) and these can also be used to evaluate oral health.

It is inferred from the above that in order to evaluate the efficacy of preventive oral health programs, the construct can be measured using these proposed models, by determining the factors that influence favorable attitudes leading to a change in people’s behavior. Instruments used to measure attitude include self-administered questionnaires. To ensure that the results of these questionnaires are reliable and valid, the validation process needs to be examined and it is here that shortcomings have been detected: many omit this rigorous and important process. Currently, no research has been reported that evaluates the validation aspects of an instrument and contributes to community prevention programs, research or the construction of questionnaires.

For that reason, it was decided to carry out the present review, which aims to analyze evidence that the validity and reliability properties of the self-administered questionnaires currently in use to gauge attitudes to oral health have been measured.

## Material and Methods

With the objectives of the research in mind, a review of the bibliography in the Medline (PubMed), SCOPUS and Web of Science (WoS) databases was carried out from 2016 to 2021. The following keywords were used in the search: (questionnaire* OR survey*) AND (attitude* OR behavior*) AND (“oral health” OR “dental care”) AND (validity OR reliability). The established inclusion criteria were articles with an observational - descriptive - cross-sectional design, where there was evidence of the use of self-administered questionnaires that measured attitude on its own or with other constructs, aimed at patients and health professionals and written in English. Those which measured attitude during the dental treatment or in a specific context, and those applied to children or patients with special conditions were excluded.

The evidence of validity and reliability of the review articles found will be cataloged according to “COnsensus-based Standards for the selection of health status Measurement INstruments” (COSMIN) (10) ( Table 1, Table 2). Two of the researchers (R-L RP and S-H JM) carried out the initial review. When there was no consensus for the choice of articles, they met together with the other two researches (D-P ME and B-V DJ). In the end, the four researchers reviewed all the selected articles.

**Table 1:**
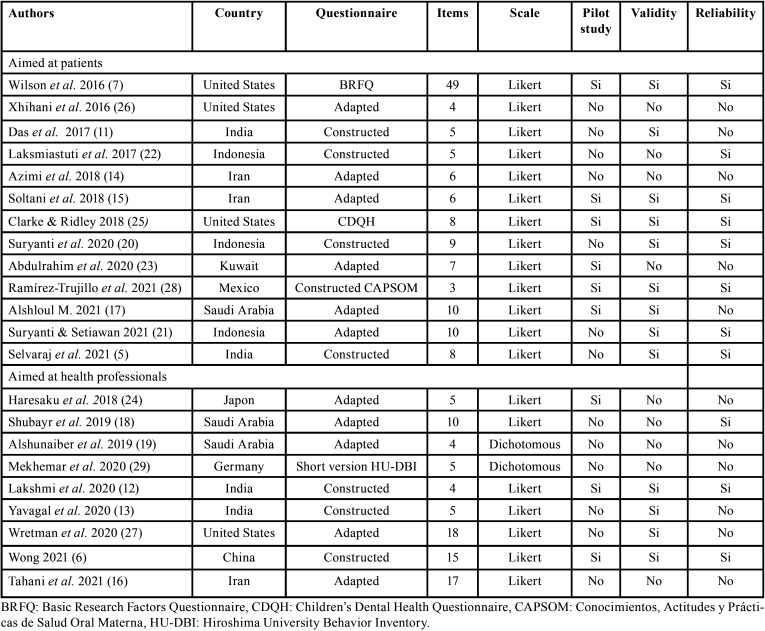
Summary of the self-administered questionnaires used to measure patients’ and health professionals attitudes to oral health.

**Table 2:**
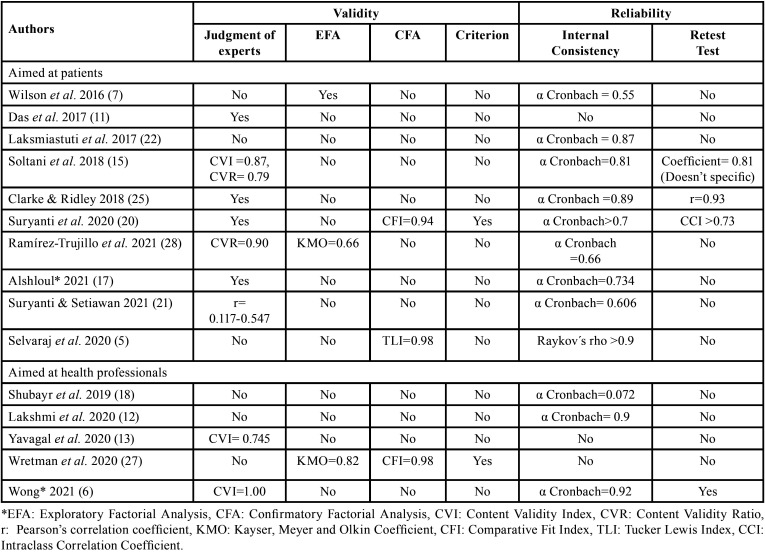
Validity and reliability aspects of self-administered questionnaires for patients and health professionals established by COSMIN criteria (10).

## Results

**Figure 1:**
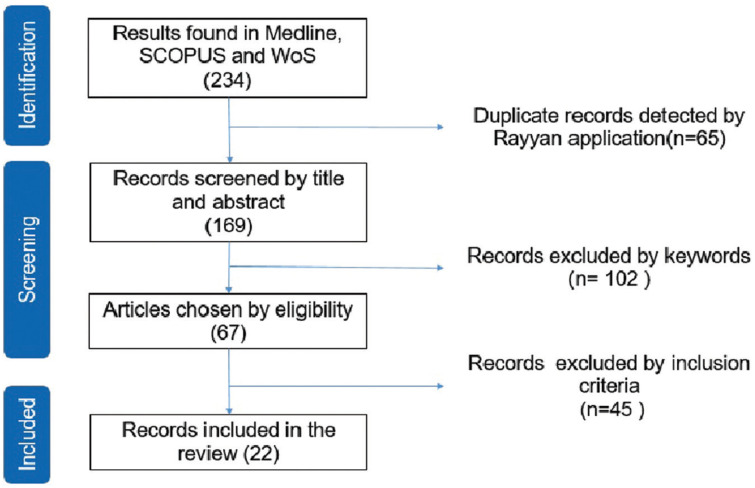
Flowchart.

Using the search strategy, a total of 234 original articles were obtained and in order to detect duplicates, a collaborative web application called “Rayyan” was used, which allowed the references to be systematized. The indexing of the 169 articles remained was confirmed in the Medline (PubMed) database. After reading their abstracts based on keywords, 67 articles were selected, of which 45 were discarded because they were systematic reviews, conference abstracts, experimental and longitudinal studies or studies in which questionnaires were answered with interviews. Of the final 22 articles (Fig. 1), 13 were aimed at patients and nine at health professionals (Table 1, Table 2).

The validation aspects of the self-administered questionnaires were evaluated using the COSMIN guide (10), which is a checklist that assesses the methodological quality of the studies and is also easy to apply. In this review, the validity property (content, criteria, construct) and the reliability property (internal consistency, test-retest, measurement error) were analyzed according to the criteria of this guide.

Most of the research studies were conducted in Asian countries (5,6,11-19,20-24). Five were carried out in North America (7,25-28) and only one in Europe (29). Of the 12 questionnaires used in the respective research studies, seven were based on theoretical models such as TPB, HBM, the cognitive-affective-conative model and COM-B model (capability, opportunity, and motivation for behavior) (5-7,15,18,19,29). Thirteen were applied in schools, universities and communities (5,7,11-13,17,20-23,25,26,29), eight in healthcare establishments (6,14-16,18,24,25,28). Of these questionnaires, one was applied in a virtual and face-to-face environment (26) and another one in a virtual environment (19).

Furthermore, of the articles that evaluated the attitude construct in patients, the pilot studies assessed apparent validity (7,17,23,28) and the validity properties and reliability (15,25) (Table 1). Additionally, six questionnaires were aimed at parents and/or caregivers of children, and evaluated their oral health attitude towards infants (7,14,15,21,22,25); three were aimed at the adult population (5,26,28) and four at adolescents (11,17,20,23). The questionnaires used to evaluate one of the dimensions of the construct attitude included the Basic Research Factors Questionnaire (BRFQ) (7) and the Children’s Dental Health Questionnaire (CDHQ) aimed at parents (25).

Of the total articles aimed at patients, eight were adapted from other studies (7,14,15,17,21,23,24,26) and one went through the process of cross-cultural adaptation from English to Arabic (17). The other five were based on questionnaires in the same language in which the research was developed.

The indices and its coefficients used to determine the validity and reliability properties of the reviewed articles are show in Table 2. It is necessary to mention that reliability in the questionnaires that evaluated attitude in patients was evaluated through internal consistency and Cronbach’s alpha coefficient was mostly used, fluctuating between 0.55 to 0.81 for the attitude subscale in six questionnaires (7,15,20,21,25,28). Three questionnaires showed the total internal consistency of the instrument (17,22,25) with coefficients of 0.87, 0.89 and 0.73 respectively (Table 2). Regarding the application of test-retest, Soltani *et al*.(15), was the only article that reported two weeks for re-application of the questionnaire (Table 2).

The items of the thirteen questionnaires referred to concepts related to attitudes towards oral hygiene, dental care, sugar consumption, dental problems, repercussions for the general health, dental pain and perception of the severity of dental caries. There were differences found in terms of content depending on the population being addressed: adolescents, parents, caregivers and pregnant women. Selvaraj *et al*. reported three dimensions: daily oral hygiene, oral hygiene habits and oral hygiene assumptions; and Wilson *et al*. took into consideration four dimensions: self-efficacy, perception of oral health practices, locus of control and HBM (5,7).

In addition, of the articles that evaluated the construct in health professionals, the reliability property evaluated with internal consistency, Cronbach’s alpha coefficient was also used. Two articles reported this coefficient to the attitude subscale (6,18) of the instrument , and one determined to total internal consistency (22). Six questionnaires, which had to be adapted (16,18,19,24,27,29), only one reported measuring internal consistency (18). The questionnaires reviewed were: three designed for dentistry, pharmacy and nursing students (12,13,29) including the short version of the Hiroshima University Behavior Inventory (HU-DBI) (29); five designed for nurses, doctors and health providers (6,18,19,24,27) and one for dentists in private practice (16). Two dimensions of the attitude construct were reported in Wretman *et al*., which were “care of residents´ teeth” and “care of own teeth” (27).

Finally, with respect to cross-cultural adaptation, Wong is not merely the only one who reports adaptations from English to Chinese, but also the only one who evaluates stability with test-retest over a period of 10 to 14 days. He does not mention the correlation coefficient (Table 2).

## Discussion

In any process of measuring variables under investigation, whether or not using questionnaires, it is important to evaluate reliability and validity. In this particular case several stages are involved from an exhaustive review and compilation of information on the construct of interest, to obtaining an instrument that is simple, viable, culturally adapted and sensitive to change, with clearly defined, reliable and valid dimensions. Psychometric models allow the dimensions of aspects of the construct to be defined, offering a new approach in the elaboration or adaptation of the questionnaire and ensuring good results with respect to the information collected on attitudes to oral health. For this reason, 20 original articles were analyzed where different self-administered questionnaires had been used to measure the construct. This is the first review report that evaluates and synthesizes measurement properties (validity and reliability) of the attitude construct in oral health.

The most important aspect of the property validity is content validity (10), which refers to analyzing the concept that it is intended to measure uses the judgment of experts. In this review, nine articles evaluate this (6,11,13,15,17,20,21,25,28), and five of them (6,13,15,21,28) report its index. Only two articles (6,17) report on cross-cultural adaptation to ensure conceptual and semantic equivalence. Moreover, criterion validity refers to the alternative method used as a reference in measuring of the construct. Two studies reported this aspect of the construct with the dental plaque index (20,25).

Construct validity relates to whether the instrument reflects the theory concerning it and can be expressed in dimensions (30,31,32). Only four reported this aspect through factor analysis using the Tucker Lewis Index (TLI), Kayser, Meyer and Olkin Coefficient (KMO) and Comparative Fit Index (CFI) (5,7,20,26). One study reported validity with the coefficient of corrected item-total correlation >0.4 (22); but this coefficient is not a measure to determine validity (33). This is a requirement to perform a factorial analysis (r between 0.3 and 0.9), which is done in the property of the construct. Therefore, we did not consider that questionnaire as a validated instrument. Other aspects such as discriminant, logical and convergent validity were only reported by Wretman *et al*. (27). Finally, the property of reliability is the consistency and stability of the measurements when the measurement process is repeated over and over again. Cronbach’s alpha values for internal consistency were reported in most studies (6,7,12,15,17,18,20-22,25,28). Evaluation of the attitude subscale of the questionnaire (6,7,15,18,20,21,28), in contrast to those that evaluated the total scale of the questionnaire (12,17,22,25), limit the chance to make comparisons between other subscales that are related to the attitude construct. Therefore, the results of this property, according to the COSMIN guide, were considered accepTable. Few studies published test-retest data (6,15,25,20). Reported coefficients of the latter are considered to be in agreement to an accepTable degree. Failure to report this measurement in most studies makes their results unreliable.

The questionnaires by Suryanti *et al*. (20) and Ramirez-Trujillo *et al*. (28) are interesting. They were aimed at patients who met the aspects of validity and reliability and included accepTable coefficients; the Hiroshima questionnaire may also be appropriate for health professionals.

A fluctuation was found in the number of items (3 to 49) and dimensions (1 to 5), but most of them were expressing similar ideas. In fact, questionnaires with a greater number of items cause fatigue among the respondents. These aspects have to be taken into consideration when conducting similar investigations or implementing these instruments in oral health programs. Self-administered questionnaires evaluated in the present review have the advantage of being cheap, easy to administer and capable of being delivered digitally. Questionnaires via online can be used in situations such as the current pandemic, but a negative fact about these self-administered questionnaires is that they can be influenced by information bias.

In conclusion, this review shows that the questionnaires used partially comply with the properties of validity and reliable measurement of the variables under investigation with regard to attitudes to oral health.
